# Nodular B-Cell Cutaneous Lymphoid Infiltrate With Limited Tissue for Molecular Evaluation: A Case Highlighting Diagnostic Challenges and Management Variability

**DOI:** 10.7759/cureus.106928

**Published:** 2026-04-12

**Authors:** Sri Naidnur, Coral Martes-Villalobos, Sonia A Neave, Emily DeSantis, Rick Lin

**Affiliations:** 1 Dermatology, Oasis Dermatology Group, McAllen, USA; 2 Dermatology, Universidad Central del Caribe School of Medicine, Bayamón, PRI; 3 Dermatology, HCA Healthcare Corpus Christi Medical Center - Bay Area, McAllen, USA; 4 Dermatopathology, Sagis Diagnostics, Houston, USA

**Keywords:** b-cell infiltrate, cutaneous lymphoid infiltrate, dermatology, dermatopathology, lymphoid infiltrate

## Abstract

Cutaneous lymphoid infiltrates can mimic lymphoma and often present a diagnostic challenge. While gene rearrangement studies may help assess clonality, they are not always feasible in routine practice. We present a case of a nodular B-cell-predominant lymphoid infiltrate in the left proximal arm for which molecular testing could not be performed due to insufficient tissue. Histopathology and immunohistochemistry favored a reactive process, but diagnostic uncertainty remained. Management differed between providers. Dermatopathology recommended clinical monitoring, while the treating dermatologist referred the patient to hematology/oncology for further evaluation. The patient underwent a systemic workup but was ultimately lost to follow-up. This case highlights the limitations of tissue sampling, variability in management, and the importance of clinicopathologic correlation in cutaneous lymphoid infiltrates.

## Introduction

Cutaneous B-cell lymphoid infiltrates are typically reactive processes that may clinically and histologically mimic primary cutaneous B-cell lymphoma, making accurate diagnosis challenging [1-3]. These infiltrates are often antigen-driven and may be triggered by arthropod bites, infections, medications, or trauma; however, many cases remain idiopathic [2,3].

Cutaneous pseudolymphoma refers to a group of benign, reactive lymphoid proliferations that may be B-cell, T-cell, or mixed and can closely mimic cutaneous lymphoma both clinically and histologically [1]. Misdiagnosis may lead to delayed recognition of malignancy or, conversely, unnecessary workup and patient anxiety when a reactive process is mistaken for lymphoma.

Clinically, they most commonly present as solitary or localized papules or nodules and can be difficult to distinguish from lymphoma, as reactive and neoplastic processes often overlap in both appearance and histology [1,4]. These lesions are most frequently seen in adults and may occur across a wide age range, with no consistent sex predilection reported [2,4]. Reactive infiltrates generally follow a benign course and may remain stable or regress, whereas primary cutaneous B-cell lymphomas represent clonal proliferations with typically indolent behavior in most subtypes, although some variants may be more aggressive [1-4].

Histopathologic evaluation with immunohistochemistry remains central to diagnosis [1,5,6]. Features such as a mixed cellular composition and polytypic plasma cells favor a reactive process, whereas monotypia and cytologic atypia raise concern for lymphoma [1-4]. In general, reactive infiltrates tend to preserve normal tissue architecture, while lymphomas more often show a more uniform (monomorphic) appearance with architectural effacement and light-chain restriction; however, overlap exists, and clinicopathologic correlation remains essential for accurate diagnosis [1-4]. Molecular studies, including gene rearrangement testing, may assist in assessing clonality [6]; however, these are not always feasible in routine practice due to limited tissue and must be interpreted in the appropriate clinical context [1,3,5,6]. 

Given this overlap, management may vary when diagnostic certainty is limited. We present a case of a nodular B-cell-predominant cutaneous lymphoid infiltrate in which molecular testing could not be performed due to insufficient tissue, highlighting diagnostic limitations and variability in real-world management.

## Case presentation

A 48-year-old female patient presented with a solitary lesion on the left proximal arm that had been present for approximately four years. The lesion initially demonstrated slow growth but had remained stable in size for several years prior to presentation. The patient reported that it was pruritic when first noticed but had since become largely asymptomatic, with only mild tenderness on palpation. She denied bleeding, ulceration, or rapid change in size. She denied B symptoms (fever, night sweats, or unintentional weight loss). The patient denied any local trauma, arthropod bite, prior injection at the site, or new medication exposure.

**Figure 1:**
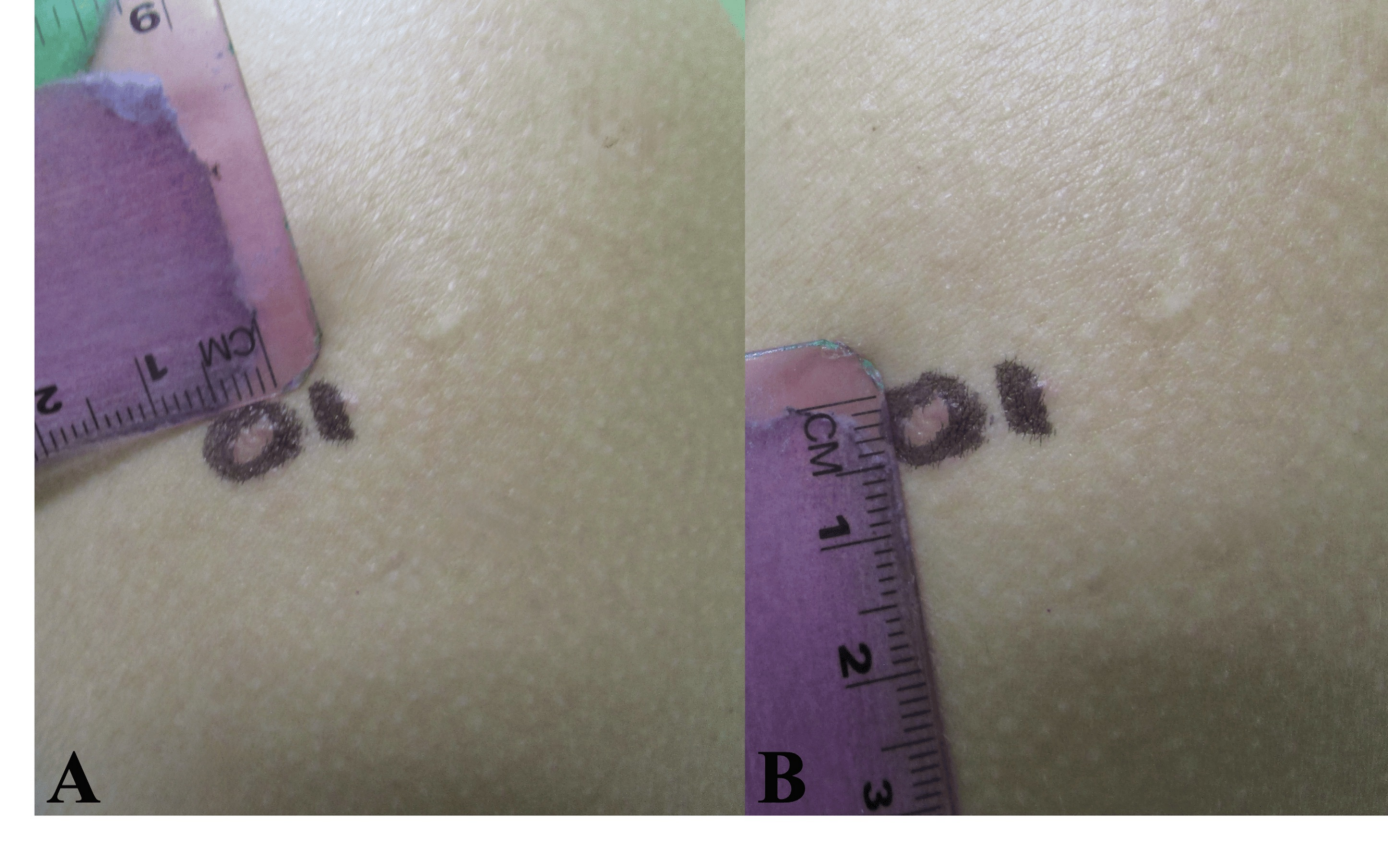
Clinical presentation. (A,B) Fitzpatrick skin type IV with a well-circumscribed, erythematous to skin-colored, slightly indurated nodule on the left proximal arm. Lesion size (~6 mm in A and ~5 mm in B) with measurement scale. The lesion was marked for a 6-mm punch biopsy.

On physical examination, there was a well-circumscribed, erythematous, slightly indurated nodule measuring approximately 6 mm x 5 mm in diameter on the left proximal arm (Figures 1A-1B). No regional lymphadenopathy was appreciated. The initial clinical differential diagnoses included cutaneous pseudolymphoma, dermatofibroma, dermatofibrosarcoma protuberans, and hypertrophic scar.

**Figure 2:**
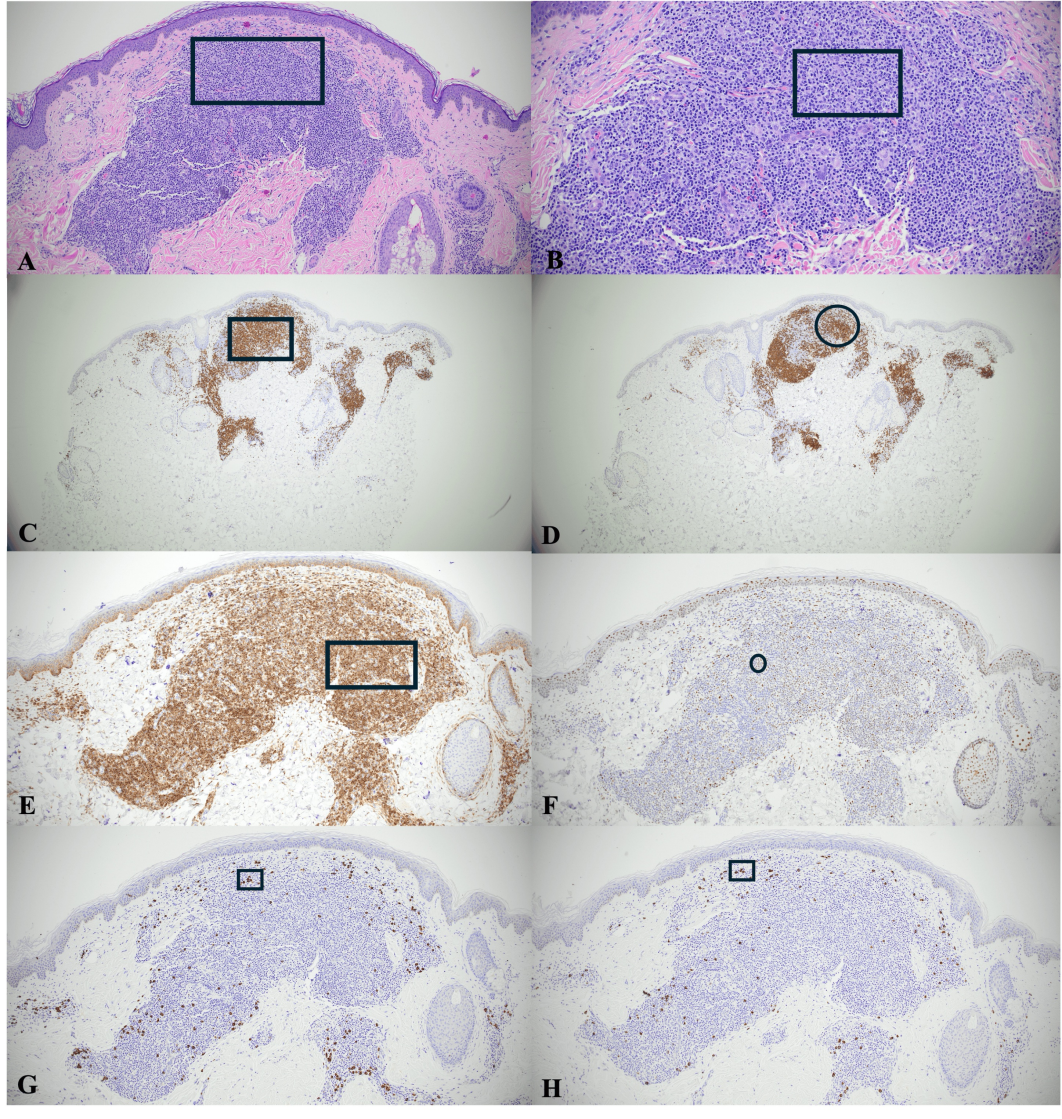
Histopathologic and immunohistochemical findings. (A) Hematoxylin and eosin (H&E)-stained section at 4× magnification showing a dense superficial dermal infiltrate of small- to medium-sized lymphocytes (rectangle highlights a representative area). (B) Similar findings at 10× magnification (rectangle highlights a representative area of the lymphocytic infiltrate). (C) CD3 immunostain highlighting T-cell-predominant areas (rectangle). (D) CD20 immunostain highlighting B-cell aggregates (circle). (E) BCL-2 expression is limited to T cells (rectangle). (F) BCL-6 highlighting scattered follicular center cells (circle). (G,H) Kappa and lambda in situ hybridization demonstrating a polytypic plasma cell population (boxes highlight representative areas).

A 6-mm punch biopsy was performed, and the lesion was entirely removed at the time of the procedure. Histopathologic examination revealed a predominantly superficial, nodular dermal infiltrate composed of small- to medium-sized lymphocytes with at least one ill-defined lymphoid follicle (Figures 2A-2B). Immunohistochemical (IHC) studies demonstrated a mixed lymphoid population, consisting of approximately 60% CD3⁺ T cells (Figure 2C) and 40% CD20⁺ B cells (Figure 2D). Although T cells were more numerous, the designation of a B-cell-predominant infiltrate was based on nodular architecture and clusters of B cells rather than overall cell count.

The CD4:CD8 ratio was within normal limits. CD21 highlighted a delicate, wispy meshwork of follicular dendritic cells. BCL-2 expression was limited to T cells (Figure 2E). A subset of B cells expressed BCL-6 (Figure 2F), while CD10 was largely negative. Plasma cells were present and demonstrated polytypic kappa and lambda (Figures 2G-2H) expression by in situ hybridization (ISH).

No significant cytologic atypia, epidermotropism, or features of overt malignancy were identified. Due to limited tissue, molecular clonality testing (gene rearrangement studies) was not performed.

Taken together, these findings favor a reactive lymphoid infiltrate rather than overt lymphoma, as evidenced by the mixed T- and B-cell composition, normal T-cell ratio, and polytypic plasma cells. Dermatopathology recommended a conservative, wait-and-watch approach with clinical monitoring for B symptoms, lesion regrowth, or development of new similar lesions.

Given the diagnostic uncertainty, the treating dermatologist elected to refer the patient to hematology/oncology for further evaluation. The patient expressed a preference for a more comprehensive evaluation and elected to proceed with this recommendation. She reported that a PET scan had been performed; however, she was subsequently lost to follow-up. At her last documented visit six months after biopsy, she denied B symptoms, regrowth of the lesion, or development of new lesions.

Overall, the findings favored a reactive process; however, limited tissue prevented molecular testing and did not fully resolve diagnostic uncertainty, requiring clinical correlation and follow-up.

## Discussion

Cutaneous lymphoid infiltrates represent a spectrum of reactive and neoplastic processes that can be difficult to distinguish, particularly in limited biopsy specimens. These infiltrates may be composed of B cells, T cells, or both, and diagnosis relies on clinicopathologic correlation rather than any single feature [1-3,5].

In this case, several findings favored a reactive process. Although T cells were more numerous, the designation of a nodular B-cell infiltrate was based on architecture rather than proportion [1,4]. B-cell aggregates with follicular features and BCL-6 expression supported a follicular center-type B-cell population, while admixed T cells represented a reactive background [1,5]. BCL-2 staining limited to T cells further supported this, as germinal center B cells are typically BCL-2 negative [1,5,6]. The mixed lymphoid population and plasma cells with polytypic kappa and lambda expression on ISH argued against clonality [2,6]. The CD4:CD8 ratio was within normal limits, without a significant imbalance. The absence of epidermotropism and the lesion’s stability over four years without growth or systemic symptoms further supported a benign process [1,2,4].

Reactive cutaneous lymphoid infiltrates, often termed cutaneous pseudolymphomas, are typically benign, antigen-driven processes associated with triggers such as arthropod bites, medications, or trauma [2,3,6]. They often present as solitary lesions and may persist for months to years, contributing to overlap with low-grade lymphoproliferative disorders [2-4].

Diagnostic certainty may be limited in routine practice. In this case, the lesion was completely removed with a 6-mm punch biopsy, leaving insufficient tissue for molecular clonality testing, a common real-world limitation [3,5]. While not always required, the absence of molecular studies may warrant cautious interpretation [6]. Accordingly, this diagnosis is best considered an interpretative or working diagnosis based on available clinicopathologic findings. In cases where additional tissue is available, further evaluation with molecular studies, flow cytometry, or repeat biopsy may be considered to help reduce diagnostic uncertainty.

This case also highlights variability in management. Dermatopathology favored observation based on benign features; however, the treating dermatologist referred the patient to hematology/oncology. The patient preferred a more comprehensive assessment and elected to proceed with this referral. Systemic workup or specialist referral may be considered in cases with diagnostic uncertainty or concern for lymphoproliferative disease [2,5]. Management is not standardized and may vary based on clinical suspicion and patient preference. This case reflects variability in real-world management and highlights the importance of individualized, clinicopathologic decision-making.

## Conclusions

Reactive nodular cutaneous B-cell lymphoid infiltrates can closely mimic primary cutaneous B-cell lymphoma both clinically and histologically, making diagnosis challenging, particularly when tissue is limited. In such cases, features such as a mixed lymphoid population, normal T-cell ratios, absence of cytologic atypia, and polytypic plasma cells may support a benign process; however, the absence of molecular studies may warrant diagnostic caution.

These entities lack standardized management guidelines, and clinical decision-making is often nuanced. This case highlights the importance of clinicopathologic correlation and illustrates how management may vary in real-world practice based on clinician judgment and patient preference. Careful follow-up remains a reasonable approach when findings favor a reactive process.
